# Psychotropic Medication Prescribing for Neuropsychiatric Comorbidities in Individuals Diagnosed with Autism Spectrum Disorder (ASD) in the UK

**DOI:** 10.1007/s10803-019-04291-8

**Published:** 2019-11-13

**Authors:** Basmah H. Alfageh, Kenneth K. C. Man, Frank M. C. Besag, Tariq M. Alhawassi, Ian C. K. Wong, Ruth Brauer

**Affiliations:** 1 Research Department of Practice and Policy, School of Pharmacy, University College London, London, UK; 2 College of Pharmacy, King Saud University, Riyadh, Kingdom of Saudi Arabia; 3 Centre for Safe Medication Practice and Research, Department of Pharmacology and Pharmacy, Li Ka Shing Faculty of Medicine, The University of Hong Kong, Pokfulam, Hong Kong; 4 Department of Medical Informatics, Erasmus University Medical Center, Rotterdam, The Netherlands; 5 East London Foundation NHS Trust, Bedfordshire, UK; 6 Maudsley Hospital & Institute of Psychiatry, Psychology and Neuroscience, King’s College London, London, UK; 7 Medication Safety Research Chair, College of Pharmacy, King Saud University, Riyadh, Kingdom of Saudi Arabia

**Keywords:** Autism spectrum disorder, Psychotropic medication, Prevalence, Incidence

## Abstract

Autism spectrum disorder (ASD) is a lifelong disorder. In the UK, risperidone is the only psychotropic medication approved for the management of the behavioural symptoms that may accompany autism. This is a population-based study aimed to provide an evaluation of the changing trend in the incidence and prevalence of ASD and to analyse the pattern of psychotropic medication prescribing in the UK. 20,194 patients with ASD were identified. The prevalence increased 3.3-fold from 0.109 per 100 persons in 2009 to 0.355 per 100 persons in 2016. Approximately one-third of the identified cohort was prescribed at least one psychotropic medication. Although the medications approved to manage the symptoms of ASD are limited, the prescribing of such medications is increasing.

**Electronic supplementary material:**

The online version of this article (10.1007/s10803-019-04291-8) contains supplementary material, which is available to authorized users.

Autism spectrum disorder (ASD) is a persistent neurodevelopmental condition characterised by social communication impairment and stereotyped, repetitive patterns of behaviour (APA 2013). A previous study in the United Kingdom (UK) using electronic primary care data showed that the incidence rate of pervasive developmental disorders (PDDs) increased from 0.40/10,000 (95% CI 0.30–0.54) to 2.98/10,000 (95% CI 2.56–3.47) person-years between 1991 and 2001 (Smeeth et al. 2004b). Another study showed that 70% of 112 children with autism had at least one neuropsychiatric comorbid disorder, of which the most common diagnosis was social anxiety disorder (29.2%; 95% CI 13.2–45.1) (Simonoff et al. 2008).

Psychotropic medications, such as antipsychotics, antidepressants, antiepileptic drugs and stimulants, have been used for ASD patients with associated comorbid conditions (National Collaborating Centre for Mental Health 2012). There is, however, limited evidence to guide psychotropic medication prescribing in the ASD population. Risperidone is the only drug approved by the European Medicines Agency (EMA), the Medicines and Healthcare Products Regulatory Agency (MHRA) of the UK, and the Australian Therapeutic Goods Administration (TGA) for the management of behavioural disturbance in children and adolescents with autism and conduct disorder (European Medicines Agency 2007; World Health Organisation 2013).

ASD can lead to impairment of the quality of life and the productivity of affected persons and their families; therefore, ASD and its complications have a significant impact on society and the individual, which should not be ignored. The aim of this study was to provide up-to-date information on the prevalence of ASD, psychiatric comorbidities and psychotropic medication prescribing in a UK population sample with individuals from all ages. We also aimed to examine the average duration of psychotropic treatment in treated patients.

## Methods

### Study Design

This is an observational descriptive study conducted to investigate the incidence and prevalence of ASD.

### Data Source

We used data from The Health Improvement Network (THIN) for this study. THIN is a primary care database of anonymised general practice records, covering a period from the early 1990s to the present day, of more than 11 million individuals, including 3.7 million active patients, which covers approximately 6% of the UK population (Fardet et al. 2017; Blak et al. 2011). THIN database is representative of the UK population (Blak et al. 2011), and it has been validated for use as a source of data for pharmacoepidemiological research (Lewis et al. 2007). Only individuals with a record that passed the validation check were included in the study (IMS Health 2015).

### Ethical Approval

Ethical approval for this fully anonymised study was obtained from the Scientific Review Committee (SRC) which was established to review research using the THIN database (Ref: 18THIN010).

### Study Population

All individuals in the THIN database from all age groups who had a diagnosis record of ASD in the study period between 1st of January 2009 and 31st of December 2016 were identified.

**Fig. 1:**
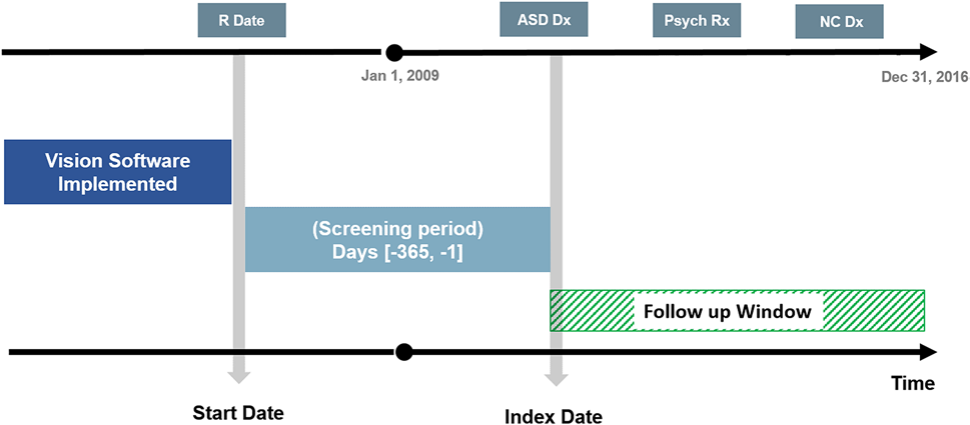
Study population. ^a^Vision software is a computerised clinical management system used by general practices to record patient information. ^b^Start date is the latest of either the date of the patient’s registration at the general practice or the date that the general practice began using vision software. *R Date* date of patient’s registration in the GP, *ASD Dx* autism spectrum disorder diagnosis, *Psych Rx* psychotropic drug prescription, *NC Dx* neuropsychiatric comorbidity diagnosis

For each patient, the latest of either the date of the patient’s registration at the general practice or the date that the general practice began using a computerised clinical management system was identified as the patient’s start date. Only patients who had an observation period of at least 12 months available from their start date were included. The date of the first recorded diagnosis of ASD following the patient’s start date was identified as the patient’s index date. Age at first recorded diagnosis was defined as the time between the birth year and the year of the diagnosis. Figure 1 illustrates the study population.

### Incident Cases Definition

For each patient, the records were screened from the start date to identify the incident cases throughout the study period (2009–2016). ASD-incident patients were defined as (1) those who had a first diagnosis of ASD following the 1st-year screening period and (2) those with an ASD diagnosis aged < 2 years during the 12-month screening period (ASD is usually diagnosed at age two or older; if the patient had a record under 2 years of age, they were counted as an incident case).

Fombonne et al. have validated the ASD Read codes list (2004); this code list has been used in other published studies carried out on UK primary care databases (Smeeth et al. 2004a, b).

Additionally, we set out to ascertain medications prescribed to people with ASD and neuropsychiatric comorbidities by using related treatment/diagnostic Read codes.

### Neuropsychiatric Comorbidities

Based on the NICE guideline on the management of ASD in individuals under 19 years of age (National Institute for Health and Care Excellence 2013) and a literature review (Simonoff et al. 2008; Murray et al. 2014), the neuropsychiatric comorbidities of interest selected were: attention deficit hyperactivity disorder (ADHD), anxiety, behavioural/conduct disorders, intellectual disabilities, sleep disorders, depression, epilepsy, schizophrenia and tic disorders. Read codes for neuropsychiatric comorbidities were obtained from the official website of the University of Cambridge (University of Cambridge) and published studies (Barnett et al. 2012; Murray et al. 2014; Hire 2016; Carey et al. 2017), and the descriptions of the codes have been reviewed by a psychiatrist in our research team.

### Psychotropic Medication

The prescriptions of the study drugs for each patient recorded on or after their index date were identified in THIN, and the annual proportions of the ASD cohort prescribed psychotropic medications were calculated for each psychotropic drug class. Drug codes were used to identify the records of psychotropic medications in the *British National Formulary* (BNF) (British Medical Association 2009), which include the following drug classes: antipsychotic, antidepressant, antiepileptic, anxiolytic, stimulant and hypnotic. Drugs included in this study are listed in Online Appendix A.

### Analysis

The annual incidence of ASD per 100 persons was calculated by dividing the number of incident cases in each year by the total number of the THIN mid-year population (MYP) of the same year multiplied by 100.

Prevalent cases of ASD were defined as all patients having an ASD diagnosis. Similar to the annual incidence, the annual prevalence of ASD per 100 was calculated by dividing the number of prevalent cases in each year by the total number of the THIN MYP of the same year multiplied by 100.

Both annual incidence and prevalence were stratified by gender and age groups: children at age 2 years or younger, 3–5-year-olds (young children), 6–12-year-olds (children), 13–17-year-olds (adolescents), 18–24-year-olds (young adults), 25–64-year-olds (adults) and ≥ 65-year-olds (elderly). It should be noted that operational definitions have been used for both incidence and prevalence; the true figures would be dependent on complete, accurate diagnosis—see discussion.

The annual proportion of ASD patients treated with psychotropic drugs was calculated by dividing the number of treated patients in each class in a year by the ASD prevalent cases in the same year multiplied by 100. A secondary analysis was performed by excluding patients with only one psychotropic drug prescription.

The Kaplan–Meier survival analysis was used to estimate the average discontinuation rate of psychotropic drugs in this cohort. The percentage of the ASD cohort having other neuropsychiatric diagnoses was calculated by dividing the number of patients having a record of each diagnosis over the total ASD cohort multiplied by 100. Analyses were performed using the Statistical Analysis System (SAS) version 9.4.

## Results

### Characteristics of the ASD Cohort

Over the study period, there were 20,194 patients with at least one recorded diagnosis of ASD, 78% of them were male. The mean age of the first recorded diagnosis among females was 14.03 (SD 11.9) years and 11.5 (SD 10.7) years among males. Further details of the study cohort are provided in Table 1.

**Table 1:** Cohort characteristics

Cohort characteristics	All	Male	Female
Number of subjects (%) with at least one diagnosis of ASD	20194 (100%)	15923 (78.9%)	4271 (21.1%)
Age at first recorded diagnosis of ASD (%)			
0–2	369 (1.8%)	307 (1.5%)	62 (0.3%)
3–5	5094 (25.2%)	4225 (20.9%)	869 (4.3%)
6–12	8601 (42.5%)	7021 (34.7%)	1580 (7.8%)
13–18	3263 (16.1%)	2325 (11.5%)	938 (4.6%)
19–24	858 (4.2%)	609 (3.0%)	249 (1.2%)
25–64	1944 (9.6%)	1389 (6.8%)	555 (2.7%)
≥ 65	65 (0.3%)	47 (0.2%)	18 (0.09%)
Neuropsychiatric comorbidities (%)			
Attention deficit hyperactivity disorder (ADHD)	2897 (14.3)	2454 (12.1)	443 (2.2)
Anxiety	3077 (15.2)	2085 (10.3)	992 (4.9)
Behavioural/conduct disorders	6208 (30.7)	5023 (24.9)	1185 (5.8)
Intellectual disabilities	2093 (10.3)	1591 (7.8)	502 (2.5)
Sleep disorders	879 (4.3)	682 (3.4)	197 (0.9)
Depression	2234 (11.0)	1459 (7.2)	775 (3.8)
Epilepsy	909 (4.5)	652 (3.2)	257 (1.3)
Schizophrenia	152 (0.7)	117 (0.5)	35 (0.2)
Tic disorders	411 (2.0)	359 (1.8)	52 (0.2)
Psychotropic medication prescribing (%)			
Antidepressant	1836 (9.0)	1218 (6.0)	618 (3.0)
Antiepileptic	585 (2.8)	418 (2.0)	167 (0.8)
Antipsychotic	814 (4.0)	614 (3.0)	200 (1.0)
Anxiolytic	237 (1.1)	162 (0.8)	75 (0.3)
Hypnotic	1894 (9.3)	1510 (7.4)	384 (1.9)
Stimulant	1163 (5.7)	1015 (5.0)	148 (0.7)

### Prevalence/Incidence of ASD Diagnoses

**Fig. 2:**
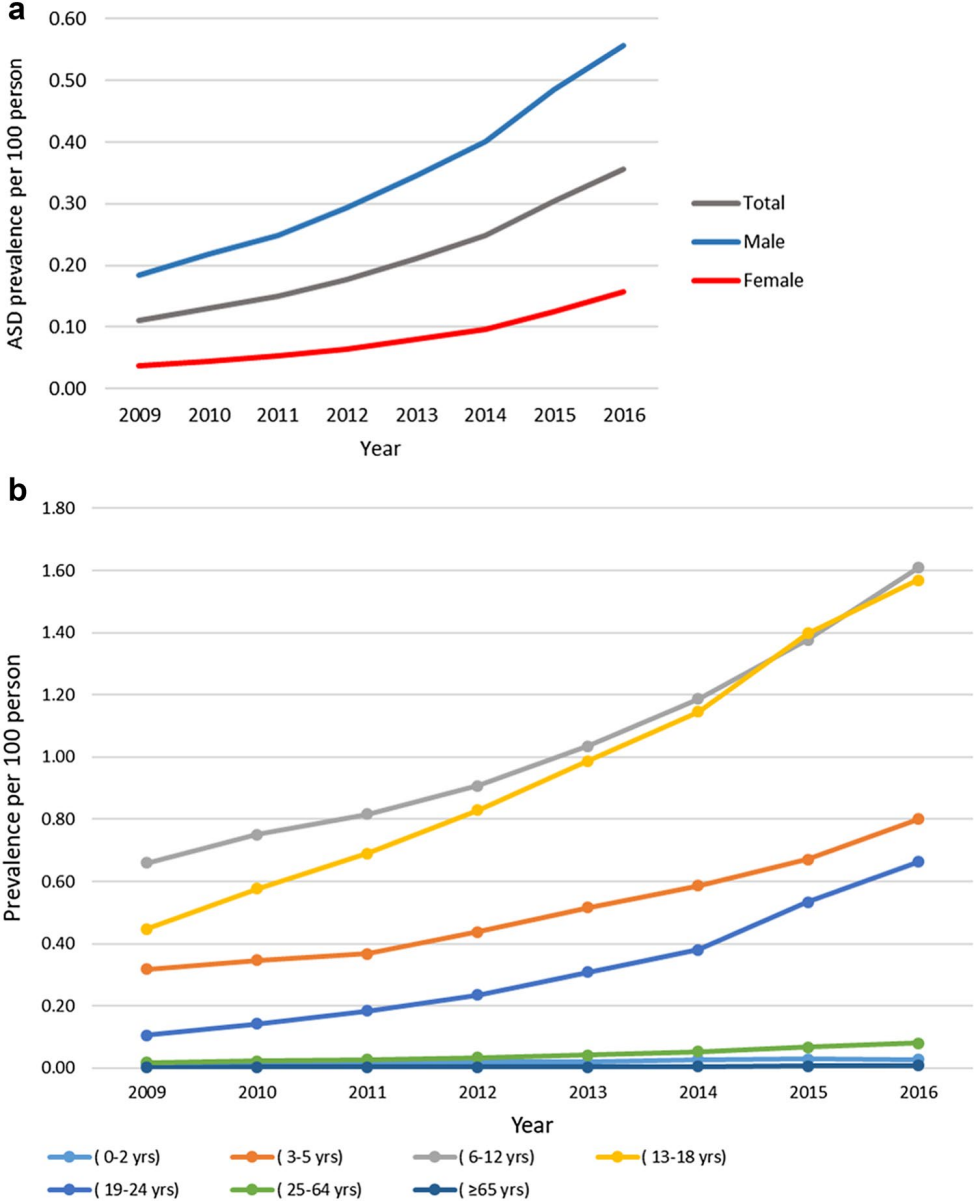
**a** ASD annual prevalence. **b** ASD annual prevalence stratified by age groups

Over the study period, the prevalence of ASD increased 3.3-fold from 0.1095 per 100 persons (95% CI 0.1094–0.1096) in 2009 to 0.3555 per 100 persons (95% CI 0.3553–0.3557) in 2016. Generally, the prevalence of ASD was higher among males than females. In 2016, the ASD prevalence was 0.1576 per 100 persons (95% CI 0.1574–0.1577) and 0.5576 per 100 persons (95% CI 0.5573–0.5580) in females and males, respectively. The prevalence of ASD was highest in individuals in the age groups of 6–12 years (children) and 13–18 years (adolescents). In 2016, the prevalence of ASD was 1.6092 (95% CI 1.6077–1.6107) per 100 persons in children and 1.5694 per 100 persons (95% CI 1.5678–1.5710) in adolescents. Figure 2a, b shows the detailed annual prevalence of ASD.

**Fig. 3:**
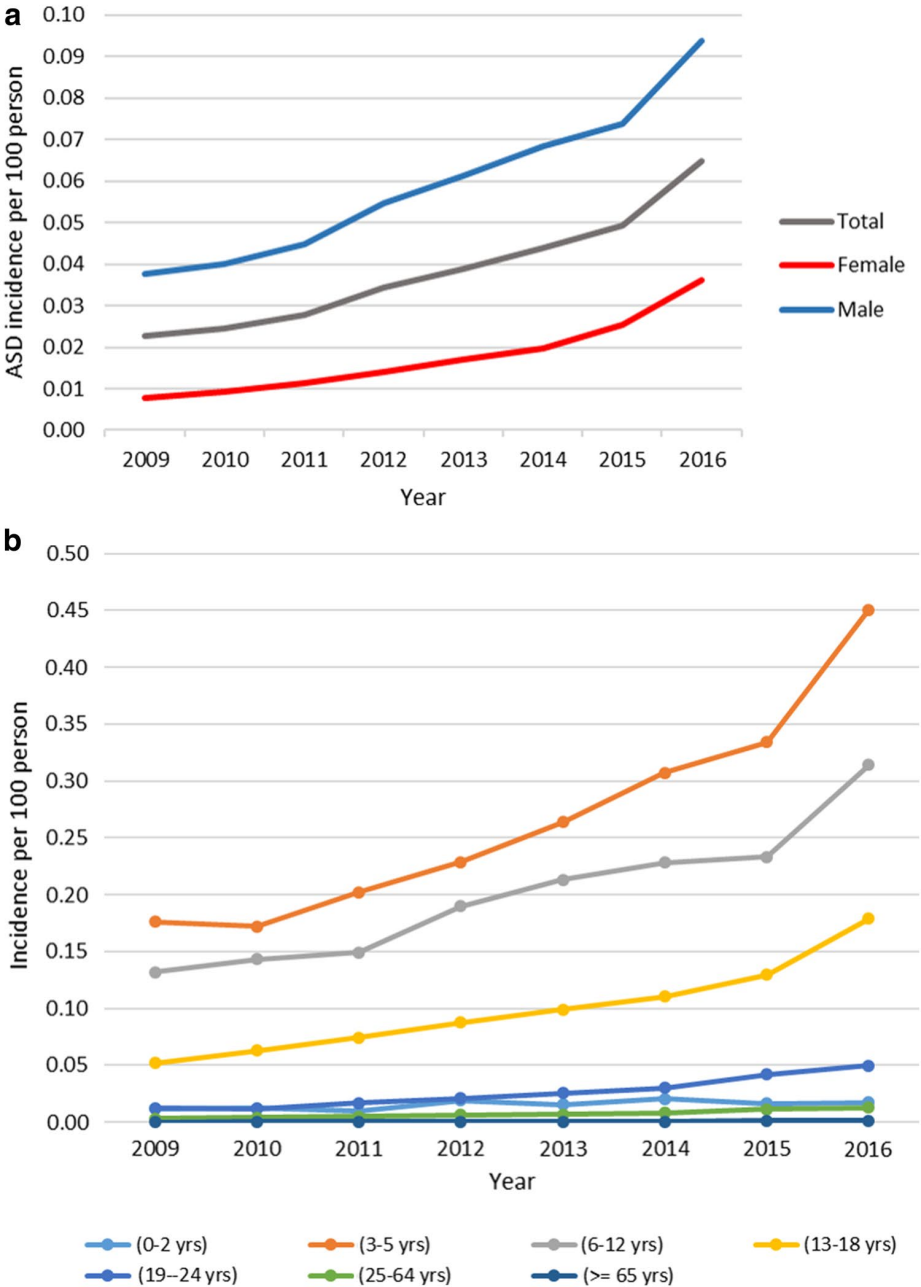
**a** ASD annual incidence. **b** ASD annual incidence stratified by age groups

The incidence of ASD rose 2.9-fold during the period 2009–2016: 0.0226 per 100 persons (95% CI 0.0226–0.0227) to 0.0647 per 100 persons (95% CI 0.0646–0.0648). The incidence rate increased 5.1-fold among females and 2.5-fold among males. During the study period, the incidence of ASD was the highest in 2016 among young children aged from 3 to 5, at 0.4505 per 100 persons (95% CI 0.4493–0.4517). The annual ASD incidence is shown in Fig. 3a, b.

### Drug Prescribing

Within the total cohort, 6529 patients (33.4%) received at least one psychotropic prescription; overall, 270,391 psychotropic prescriptions were issued. The prescribing rate was significantly greater among females (37.2% of the female cohort) compared to males (31.0% of the male cohort), *p *< 0.0001. Among the males, the three most commonly prescribed psychotropic drugs were hypnotics (in 30.5% of treated males), antidepressants (24.6%) and stimulants (20.5%). Whereas in the females, the three most commonly prescribed psychotropic drugs were antidepressants (38.8%), hypnotics (24.1%) and antipsychotics (12.5%).

The highest numbers of prescriptions issued throughout the study period were for methylphenidate (46,393 prescriptions were identified, which comprised 17.1% of all psychotropic drug prescriptions), followed by melatonin (38,520 prescriptions, 14.2%) and risperidone (19,800 prescriptions, 7.3%). Valproic acid was the most frequently prescribed antiepileptic drug (17,271 prescriptions, 6.3%). The most commonly prescribed antidepressants were fluoxetine (15,252 prescriptions, 5.6%) and sertraline (14,997 prescriptions, 5.5%).

**Fig. 4:**
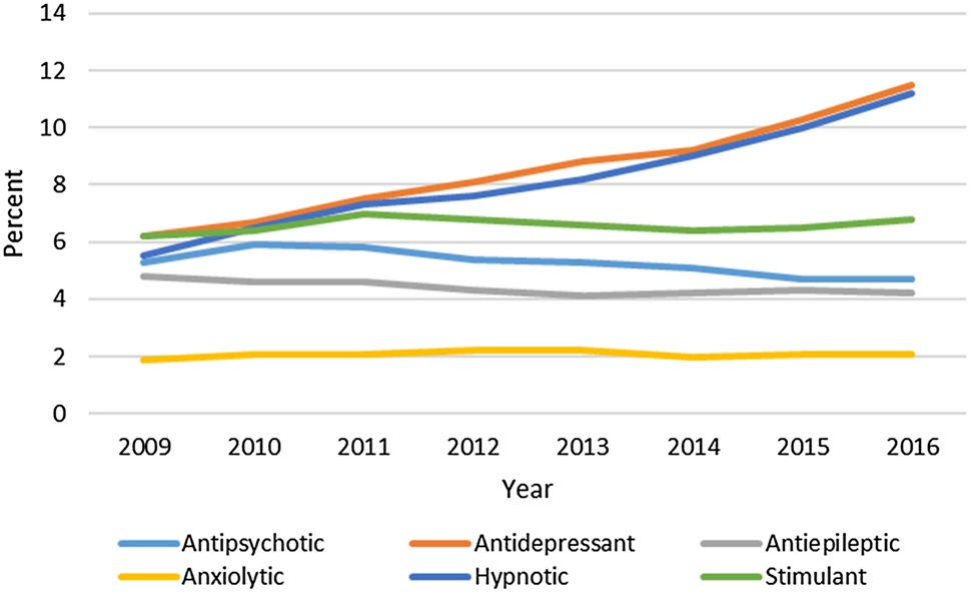
Annual percentage of psychotropic drug users per ASD cohort

The percentage of patients prescribed both antidepressant and hypnotic drugs approximately doubled over the period from 2009 to 2016: from 6.2% (95% CI 5.6–6.8) to 11.5% (95% CI 10.9–12.1) and from 5.5% (95% CI 5.0–6.2) to 11.2% (95% CI 10.6–11.8), respectively. For the remaining psychotropic drug classes, the percentage of patients prescribed these medications remained relatively steady over the study period. A secondary analysis conducted by excluding patients with only one prescription for each drug class resulted in similar findings to those of the primary analysis. Figure 4 shows the annual percentage of those prescribed psychotropic drugs in the total ASD cohort.

**Fig. 5:**
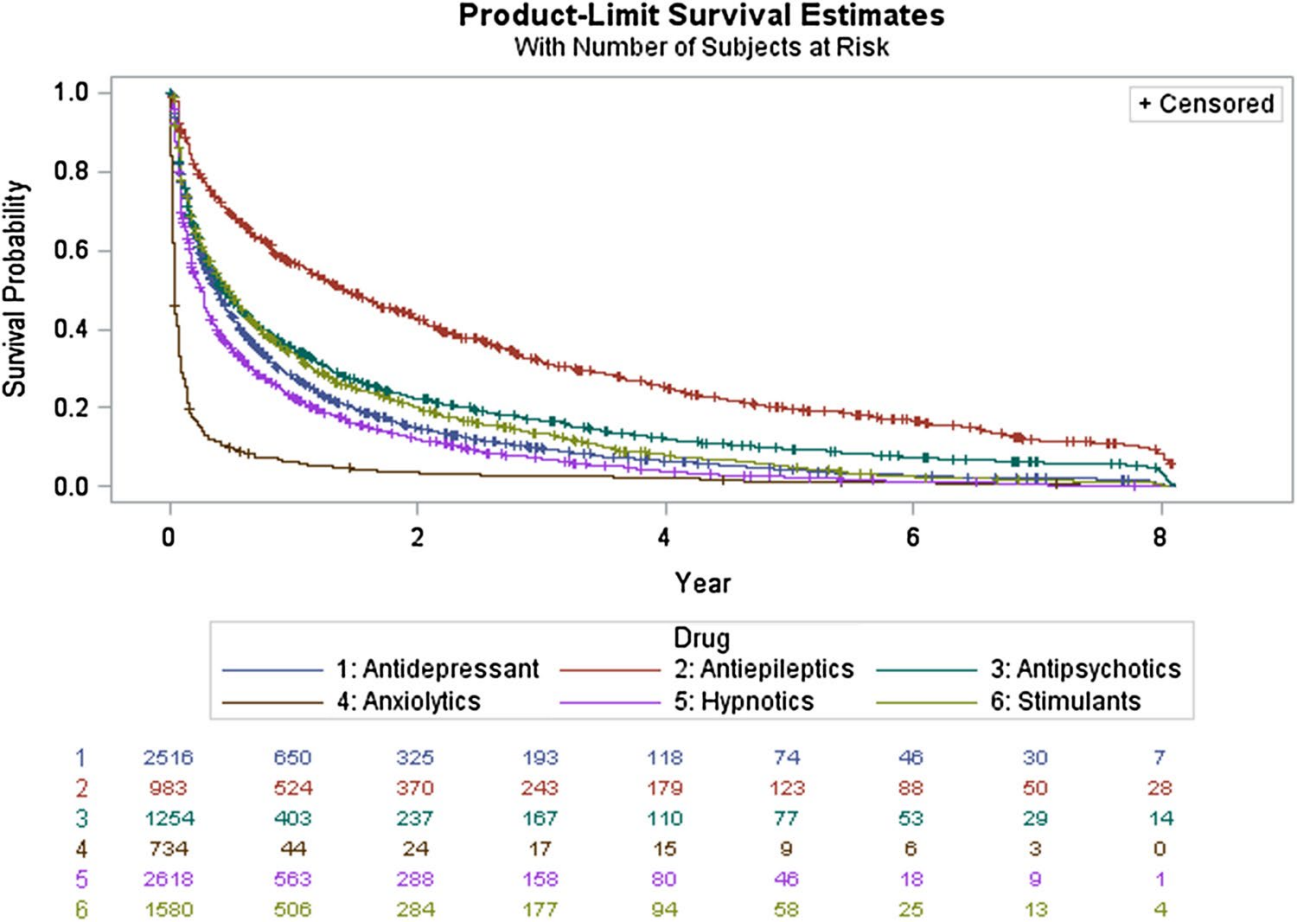
Survival analysis curves for psychotropic drug

Kaplan–Meier survival curves demonstrated that approximately one-third of those prescribed antipsychotic drugs, 403 patients of 1254 (32.1%), were on continuous antipsychotic therapy for more than 1 year, and 6.1% (77 patients) continued for up to 5 years. Furthermore, 29.9% (190 of 634) remained on risperidone therapy for more than 1 year, and 31.5% of those prescribed aripiprazole remained on the treatment for more than 1 year. For antidepressants, almost a quarter of the patients (25.8%; 650 of 2516 patients) continued on therapy for more than a year, while 2.9% of them remained on antidepressants for more than 5 years. 25% of patients prescribed fluoxetine remained on the treatment for more than 1 year. More than half of the patients prescribed antiepileptics continued on therapy for 1 year, and 12.5% of them continued for more than 5 years. The majority of ASD patients prescribed anxiolytic medication (94%) had stopped the treatment after 1 year. For patients treated with hypnotics and stimulants, 21.5% and 32% continued treatment for up to 1 year, respectively. The detailed survival analysis for psychotropic drugs identified in this study is illustrated in Fig. 5.

### Neuropsychiatric Comorbidities

Of the total ASD cohort, 57.3% of the patients had at least one neuropsychiatric comorbidity. The most commonly prescribed psychotropic drug classes in these patients were antidepressants (14.3%) and hypnotics (10.5%). The three most common neuropsychiatric diagnoses accompanying ASD were behavioural/conduct disorders at 30.7% (95% CI 30.1–31.3), anxiety at 15.2% (95% CI 14.7–15.7) and ADHD at 14.3% (95% CI 13.8–14.8). The percentage of diagnoses was higher in males than females for ADHD (15.4% and 10.3%) and behavioural/conduct disorders (31.5% and 27.7%). However, the proportion of patients with anxiety was greater among females, at 23.2% compared to 13.1% in males. Almost twice as many females (18.1%) than males (9.1%) were diagnosed with depression. For the remaining neuropsychiatric diagnoses, the proportions were similar for both males and females. Table 1 provides a full description of the numbers of neuropsychiatric diagnoses.

Among ASD patients on antipsychotics, more than half (53.3%) were diagnosed with behavioural/conduct disorders and 34.9% as having anxiety, whereas 8.1% were diagnosed with no additional neuropsychiatric comorbidities identified. Among the patients with ASD but no comorbid neuropsychiatric diagnoses, 87.6% of them were not on any psychotropic therapy; of the remaining 12.4%, 7.7% were prescribed hypnotics and 2% were on antidepressants. In those who had neuropsychiatric comorbidities, 52.8% were not on psychotropic therapy, although almost 60% of these patients had behavioural/conduct disorders.

## Discussion

### Main Findings

This study has extended the previous findings of research in this area by (1) providing the most recent and comprehensive description of ASD in the UK, (2) analysing a broader cohort, including patients with ASD of all age groups, and (3) using survival analysis to examine the duration of treatment for different psychotropic drug classes over the study period.

The incidence and prevalence of ASD have increased markedly over recent years. This probably reflects the current broader diagnostic criteria for ASD and increasing awareness of the condition. In our study, the incidence and prevalence of ASD increased 2.9-fold and 3.3-fold, respectively, from 2009 to 2016. During the study period, the greatest prevalence of ASD was seen in children (6–12-year-olds), followed by adolescents (13–18-year-olds) and then young children (3–5-year-olds). Similar trends were observed in the study by Murray et al. which covered the period from 1992 to 2008 (2014). However, the increase in the incidence and prevalence of ASD from 1992 to 2008 were much higher in Murray’s study (23.7- and 64.6-fold) compared to our findings. This could be because, by 2009, when our study period started, the broader diagnostic criteria of ASD were already well established and the level of parental and societal awareness of the condition had increased. In the US, the prevalence of ASD in 2014 in children aged 8 years was 1.68 per 100 children. This was comparable to our findings in 2014: the prevalence was 1.1870 per 100 in children aged 6–12 (95% CI 1.1859–1.1881). A 3.7:1 male-to-female ratio of ASD prevalence was identified in our study. This is similar to what was found in a prevalence study conducted in Canada over the period 2004–2015 (Hamad et al. 2019).

Although in our study there were no restrictions on age, the findings regarding psychotropic drug prescribing were consistent with the findings of Murray et al. (2014), in which, of the total cohort, 28.7% were on psychotropic therapy, compared with 32.3% in our study. In both studies the prescribing rate was higher among females than males. Furthermore, over the two study periods, from 1992 to 2008 and from 2009 to 2016, the three most commonly prescribed drugs remained the same: methylphenidate, risperidone and melatonin. This prescribing pattern corresponds with the most common comorbid neuropsychiatric diagnoses which were recognised: 14.3% of the total cohort were found to have a record of an ADHD diagnosis and 30.7% had a record of behavioural disorders. Nevertheless, the percentage of patients recorded as having a sleep disorder (4.3%) was less than what was expected based on the high rate of hypnotic drug prescribing (24.1% of treated females and 30.5% of treated males), which may be due to the under-recording of sleep disorder diagnoses. In studies that have specifically examined sleep disorders in children with ASD, the rate is usually very high compared to typically developing children, ranging from 40 to 80% (Devnani and Hegde 2015). In our study, some of the patients may have unrecorded comorbidities or comorbidities that were not identified (per protocol) in this cohort.

Aripiprazole and risperidone are antipsychotic drugs shown to be effective in the management of behavioural symptoms in children and adolescents with ASD (Owen et al. 2009; Shea et al. 2004). In two multinational studies investigating the treatment pattern of ASD, risperidone was the most commonly prescribed drug in most of the countries involved (Hsia et al. 2014; Wong et al. 2014). The rate of psychotropic drug prescribing was higher in the US compared to the UK (approximately two-thirds of the total ASD cohort had at least one psychotropic drug prescription compared to one-third of our cohort) (Spencer et al. 2013; Houghton et al. 2017). In the US, both risperidone and aripiprazole are approved for the management of behavioural disorders accompanying autism in children, while in the UK, risperidone is the only antipsychotic drug which has been approved for the same indication. Although aripiprazole has not yet been approved in the UK for the treatment of ASD, in our cohort almost 2% of psychotropic medication prescriptions were for aripiprazole (5241 aripiprazole prescriptions were issued). Moreover, 31.5% of patients treated with aripiprazole remained on the drug for more than 1 year. A study conducted in a secondary care setting in the UK found that over a 6-year observation period, 10% of 3482 children with ASD and aged below 18 were on antipsychotic therapy: 55% (*n *= 191) on risperidone and 32% (*n *= 112) on aripiprazole (Downs et al. 2016).

Nearly half of the ASD patients identified in this study had at least one comorbid neuropsychiatric diagnosis, and approximately 25.1% of the total cohort had a record of two or more neuropsychiatric comorbid diagnoses. Psychotropic medication was prescribed to 12.4% of individuals with ASD but without a record of comorbid neuropsychiatric conditions, which may suggest either the under-recording of the neuropsychiatric diagnoses or the use of psychotropic drugs to treat other issues.

### Strengths and Limitations

Although the information provided by THIN database is generalisable to the UK population it only includes information for patients in primary care. Prescriptions produced by non-primary care settings, such as hospital discharge prescriptions and prescriptions provided by specialised centres, are not recorded in THIN. Prescriptions for some off-label drugs and controlled drugs may not be recorded either. This may lead to an underestimation of prescription rates. Furthermore, the database does not directly link prescriptions for drugs with their indication for use (whether this use is licensed or unlicensed). Because of this, it is not possible to determine whether recorded drugs were being prescribed to treat neuropsychiatric comorbidities of ASD. Last, information on patient compliance and adherence to the prescribed medication cannot be obtained from the database; hence, we are not certain if the patients prescribed any of the drugs were taking them correctly, if at all.

## Conclusions

Reported ASD prevalence has been increasing over time; in our study population, it reached 1.6 per 100 children in 2016. One-third of the identified cases with ASD were on psychotropic medication. 12.4% of the treated cohort were prescribed antipsychotic drugs, of which 50.7% was risperidone and 49.3% was other antipsychotic medication, the latter having been “off-label”. This suggests that the guidance on psychotropic prescribing in children with ASD needs to be reviewed. We recommend further studies to obtain better data on the efficacy, tolerability and safety of psychotropic medication in patients with ASD, particularly children.

## Electronic Supplementary Material

Below is the link to the electronic supplementary material.

Supplementary material 1 (PDF 41 kb)
