# Supplementary material for: Psychotropic Medication Prescribing for Neuropsychiatric Comorbidities in Individuals Diagnosed with Autism Spectrum Disorder (ASD) in the UK
Source: J Autism Dev Disord. 2019 Nov 13;50(2):625–33. doi: 10.1007/s10803-019-04291-8 (PMC6994549; doi:10.1007/s10803-019-04291-8)
Supplement: Supplementary file 1 — Supplementary material 1 (PDF 41 kb) [file 10803_2019_4291_MOESM1_ESM.pdf]

Journal:
Journal of Autism and Developmental Disorders.

Title:
Psychotropic Medication Prescribing for Neuropsychiatric Comorbidities in Individuals Diagnosed with Autism Spectrum Disorder (ASD) in the UK

Authors:
Basmah H Alfageh; Kenneth K. C. Man; Frank MC Besag; Tariq M Alhawassi; Ian CK Wong; Ruth Brauer

Corresponding author:
Dr. Ruth Brauer, PhD.

Research Department of Practice and Policy, School of Pharmacy University College London

BMA House Tavistock Square London WC1H 9JP, London, UK

E-mail: r.brauer@ucl.ac.uk

Tel: +44(0)20 7874 1273

Appendix A. Psychotropic medication list

Antipsychotics	Antidepressants	Hypnotics	Anxiolytics	Stimulants	Antiepileptics
Amisulpride	Agomelatine	Chloral	Alprazolam	Amphetamine	Acetazolamide
Aripiprazole	Amitriptyline	Clomethiazole	Bromazepam	Atomoxetine	Beclamide
Asenapine	Amoxapine	Cloral betaine	Buspirone	Caffeine	Brivaracetam
Benperidol	Bolvidon	Clorazepate	Chlordiazepoxide	Dexamfetamine	Carbamazepine
Chlorpromazine	Butriptyline	Dichloralphenazone	Chlordiazepozide	Dexedrine	Clobazam
Chlorprothixene	Citalopram	Flunitrazepam	Chlormezanone	Guanfacine	Clonazepam
Clozapine	Clomipramine	Flurazepam	Clobazam	Lisdexamfetamine	Diazepam
Dartalan	Desipramine	Loprazolam	Clorazepate dipotassium	Methylperidate	Eslicarbazepine
Droperidol	Dosulepin	Lormetazepam	Diazepam	Methylphenidate	Ethosuximide
Flupentixol	Dothiepin	Mandrax	Generic Kalms tablets	Modafinil	Fosphenytoin
Fluphenazine	Doxepin	Melatonin	Ketazolam	Pemoline	Gabapentin
Haldol	Duloxetine	Methyprylon	Lorazepam	Reactivan tab	Gardenal
Haloperidol	Escitalopram	Nitrados	Medazepam	Ronyl	Lacosamide
Levomepromazine	Fluoxetine	Nitrazepam	Meprobamate	Sodium oxybate	Lamotrigine
Loxapine	Flupentixol	Potassium bromide	Oxazepam	Tafamidis	Levetiracetam
Lurasidone	Fluvoxamine	Promethazine	Prazepam		Mesuximide
Olanzapine	Imipramine	Sodium oxybate	Serenid forte		Methsuximide
Oxypertine	Iprindole	Temazepam			Methylphenobarbital
Paliperidone	Iproniazid	Triazolam			Midazolam
Pericyazine	Isocarboxazid	Triclofos			Ospolot
Perphenazine	Lofepramine	Zaleplon			Oxcarbazepine
Pimozide	L-tryptophan	Zolpidem			Paraldehyde
Promazine	Maprotiline	Zopiclone			Paramethadione
Quetiapine	Merital				Pentamidine
Remoxipride	Mianserin				Perampanel
Risperidone	Mirtazapine				Phenobarbital
Sertindole	Moclobemide				Phenobarbitone
Sulpiride	Nefazodone				Phenytoin

Thiopropazate	Nomifensine				Pregabalin
Thioproperazine	Nortriptyline				Primidone
Thioridazine	Paroxetine				Retigabine
Trifluoperazine	Phenelzine				Rufinamide
Trifluperidol	Protriptyline				Stiripentol
Zotepine	Reboxetine				Sulthiame
Zuclopenthixol	Sertraline				Tiagabine
	Sinequan				Topiramate
	Tofranil				Valproic acid
	Tranylcypromine				Vigabatrin
	Trazodone				Zarontin
	Trimipramine				Zonisamide
	Tryptophan				
	Venlafaxine				
	Viloxazine				
	Vortioxetine				
	Zimelidine				
